# Experimental Infection of Cattle with SARS-CoV-2

**DOI:** 10.3201/eid2612.203799

**Published:** 2020-12

**Authors:** Lorenz Ulrich, Kerstin Wernike, Donata Hoffmann, Thomas C. Mettenleiter, Martin Beer

**Affiliations:** Friedrich-Loeffler-Institut, Insel Riems, Germany

**Keywords:** SARS-CoV-2, COVID-19, respiratory infections, severe acute respiratory syndrome coronavirus 2, coronavirus disease, zoonoses, viruses, coronavirus, Bos taurus, cattle, experimental infection, serology

## Abstract

We inoculated 6 cattle with severe acute respiratory syndrome coronavirus 2 and kept them together with 3 uninoculated cattle. We observed viral replication and specific seroreactivity in 2 inoculated animals, despite high levels of preexisting antibody titers against a bovine betacoronavirus. The in-contact animals did not become infected.

After spilling over from an unknown animal host to humans, a novel betacoronavirus called severe acute respiratory syndrome coronavirus 2 (SARS-CoV-2) emerged in December 2019 (*1*,*2*) and induced a global pandemic. This virus, which causes coronavirus disease, was first identified in humans in Wuhan, China (*3*). The role of livestock and wildlife species at the human-animal interface in disease emergence and dynamics was extensively discussed, focusing on the identification of susceptible species, potential reservoirs, and intermediate hosts.

Natural or experimental infections have demonstrated the susceptibility of fruit bats (*Rousettus aegyptiacus*), ferrets, felids, dogs, and minks to the virus; however, pigs, chicken, and ducks are not susceptible (*4*–*6*). Besides ducks, chicken, and pigs, other major livestock species, including >1.5 billion cattle (*Bos taurus*), live with close contact with humans. Non-SARS-CoV-2 betacoronaviruses are widespread in bovines (*7*); seroprevalences reach up to 90% (*8*), but these infections are usually subclinical (*7*). However, whether any ruminant species are susceptible to SARS-CoV-2 infection or whether there is any cross-reactivity of antibodies against bovine coronaviruses (BCoVs) and SARS-CoV-2 is unknown. We examined the susceptibility of cattle to SARS-CoV-2 infection and characterized the course of infection.

## The Study

From a group of 9 dairy calves, we intranasally inoculated 6 with 1 x 10^5^ 50% tissue culture infectious dose of SARS-CoV-2 (strain 2019_nCoV Muc-IMB-1). We reintroduced the other 3 SARS-CoV-2–naïve (hereafter in-contact) cattle to the 6 infected animals 24 hours after inoculation. We monitored body temperature and clinical signs daily. We also obtained and processed blood samples and nasal, oral, and rectal swab samples (Appendix). The experimental protocol was assessed and approved by the ethics committee of the State Office of Agriculture, Food Safety, and Fisheries in Mecklenburg–Western Pomerania, Germany (permission no. MV/TSD/7221.3–2-010/18).

Before infection, all animals tested negative for SARS-CoV-2 RNA in nasal, oral, and rectal swab samples and antibodies against SARS-CoV-2 in serum samples. Veterinarians conducted daily physical examinations and noted that none of the animals (inoculated or not) showed signs of clinical SARS-CoV-2 infection (Appendix). Throughout the study, the animals’ body temperatures, feed intake, and general condition remained within normal limits (Appendix). 

We demonstrated viral replication in 2 of the inoculated animals. One animal (no. 776) tested positive for viral RNA in the nCoV IP4 real-time reverse transcription PCR (RT-PCR) on days 2 (quantification cycle [Cq] value 29.97) and 3 (Cq 33.79) after infection. Another calf (no. 768) tested positive on day 3 (Cq 38.13) (Figure, panel A). We confirmed the results with a second real-time RT-PCR selective for the *E* gene; we measured Cq values of 29.26 (no. 776, day 2 after infection), 32.12 (no. 776, day 3), and 36.18 (no. 768, day 3). We verified the results with real-time RT-PCR using the ID GENE SARS-COV-2 DUPLEX kit (IDvet, https://www.id-vet.com) (Cq values 29.17 [no. 776, day 2 after infection], 30.55 [no. 776, day 3], and 36.07 [no. 768, day 3]). These animals tested positive only in the nasal swab samples.

**Figure:**
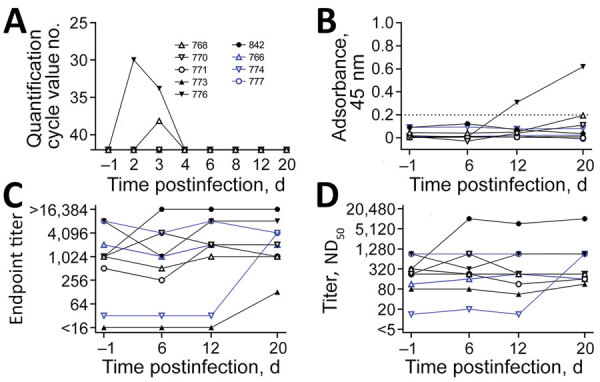
Characterization of SARS-CoV-2 infection in cattle. Animals directly inoculated shown in black. In-contact animals shown in blue. Individual animals are indicated by the same symbol in every figure panel. A) Viral load in nasal swab samples measured by real-time RT-PCR. Animals 776 and 768 had detectable viral loads on days 2 and 3 (no. 776) or day 3 only (no. 768). B) Results of indirect ELISA specific to the SARS-CoV-2 receptor binding domain. Serum samples taken on days −1 before infection and 6, 12, and 20 days after infection. Values below the dashed line are considered negative for antibodies against SARS-CoV-2. C) Results of indirect immunofluorescence assay for BCoV. D) Results of virus neutralization test for BCoV. Indirect immunofluorescence and virus neutralization test showed that animal 842, which tested positive for BCoV in the nasal swab sample by real-time RT-PCR, had an increase in antibody titer against BCoV. Preinfection antibody titers against BCoV did not affect infection with SARS-CoV-2, as animals 776 and 768, which tested positive for SARS-CoV-2, showed no infection-related reaction of BCoV antibody titers. BCoV, bovine coronavirus; ND_50_, 50% neutralizing dose, RT-PCR, reverse transcription PCR; SARS-CoV-2, severe acute respiratory syndrome coronavirus 2.

We tested serum samples with an indirect ELISA specific to the SARS-CoV-2 receptor binding domain (RBD-ELISA). An increase in seroreactivity was observed for animal 776 from day 12 onward, indicating seroconversion (Figure, panel B). On day 20, we took serum samples that confirmed the positive ELISA findings and used an indirect immunofluorescence assay (iIFA) to measure a low antibody titer of 1:4. In addition, a virus neutralization test (VNT) (serum dilution 1:2) showed a visible, although incomplete, inhibition of viral replication. Samples taken on day 20 from animal 768 showed only slightly increased seroreactivity in ELISA, whereas iIFA and VNT results remained negative. These differences might be attributable to varying test sensitivities or a possible restriction of viral replication to the upper respiratory tract. Throughout the study, the other animals tested negative for antibodies against SARS-CoV-2 by ELISA, iIFA, and VNT.

We also tested the BCoV status of each calf. Before SARS-CoV-2 infection, all animals had neutralizing antibodies against BCoV, although the titers differed substantially among individual animals (Figure, panel D). Three animals showed an increase in antibody titers against BCoV by iIFA (no. 842 and 773, which were directly infected with SARS-CoV-2, and no. 774, an in-contact animal) and 2 also by VNT (no. 842 and 774) within the study period (Figure). To show that this increase was caused by a natural BCoV infection and not SARS-CoV-2, we tested nasal swab samples for BCoV using RT-PCR selective for the *RdRp* region (*9*). Animal 842 tested positive by PCR for BCoV RNA 1 day before our experimental SARS-CoV-2 infection and 2 days after infection. We used Sanger sequencing to confirm the BCoV infection, which had increased the titer of antibodies against BCoV in this animal (Figure). Animal 842 presumably infected animal 774 with BCoV. However, we did not observe any cross-reactivity of the bovine coronavirus with the applied SARS-CoV-2 tests, because all animals tested negative by the nCoV IP4 PCR for SARS-CoV-2, the iIFA and VNT specific to SARS-CoV-2, and the RBD-ELISA (Figure) before infection. Moreover, 2 animals (nos. 776 and 768) with high BCoV seroreactivity tested positive for SARS-CoV-2 RNA after inoculation, whereas those with lower BCoV-specific titers could not be infected, further confirming a lack of any cross-reactivity or cross-protection. 

## Conclusions

Our findings demonstrate that under experimental conditions cattle show low susceptibility to SARS-CoV-2 infection. This finding corresponds with a predicted medium susceptibility of cattle species on the basis of a computational modelling of their angiotensin-I-converting enzyme 2, the cellular receptor for SARS-CoV-2 (*10*).

We inoculated 6 cattle with SARS-CoV-2; of these animals, 2 later tested positive for the virus in PCR of nasal swab samples and show specific seroconversion by RBD-ELISA. Even though the genome loads detected in animal 768 at day 3 were low, there is evidence that this animal was confronted with real viral replication. RNA residues from inoculation are only detectable shortly after inoculation; here, the day 2 nasal swab tested repeatedly PCR negative. Furthermore, other studies using the same infection dose and vaporization device also found no residual RNA on day 2 (*5*). In addition, the low-level viral replication led to a slight, but detectable, serologic reaction in the applied ELISA (Figure, panel B). 

In our study, we did not observe intraspecies transmission to in-contact cattle. Thus, we have no indication that cattle play any role in the human pandemic, and no reports of naturally infected bovines exist. Nevertheless, in regions with large cattle populations and high prevalence of SARS-CoV-2 infection in humans, such as the United States or countries in South America, close contact between livestock and infected animal owners or caretakers could cause anthropo-zoonotic infections of cattle, as has been already described for highly susceptible animal species such as minks, felids, and dogs (*6*,*11*). When assessing the risk for virus circulation within bovine populations, one should consider the age, husbandry practices, and underlying health conditions of the animals. Outbreak investigations might include cattle, particularly if direct contact has occurred between animals and persons infected with SARS-CoV-2. In addition to direct detection by PCR, serologic screenings with sensitive and specific ELISAs should also be taken into consideration. In this context, the wide distribution of BCoV is of special interest, especially because the presence of a preexisting coronavirus did not protect from infection with another betacoronavirus in this study. Double infections of individual animals might lead to recombination events between SARS-CoV-2 and BCoV, a phenomenon already described for other pandemic coronaviruses (*12*). A resulting chimeric virus, comprising characteristics of both viruses, could threaten human and livestock populations and should therefore be monitored.

This article was preprinted at https://www.biorxiv.org/content/10.1101/2020.08.25.254474v1.

Appendix Additional information on experimental design, clinical examination, and sample processing related to experimental SARS-CoV-2 infection of cattle.
