# Supplementary material for: Experimental Infection of Cattle with SARS-CoV-2
Source: Emerg Infect Dis. 2020 Dec;26(12):2979–81. doi: 10.3201/eid2612.203799 (PMC7706945; doi:10.3201/eid2612.203799)
Supplement: Appendix — Additional information on experimental design, clinical examination, and sample processing related to experimental SARS-CoV-2 infection of cattle. [file 20-3799-Techapp-s1.pdf]

Article DOI: https://doi.org/10.3201/eid2612.203799

Experimental Infection of Cattle with SARS-CoV-2

Appendix

Experimental Design and Clinical Examination

Six 4–5 month-old, male Holstein-Friesian dairy calves were intranasally inoculated under BSL-3 conditions with 1×10^5 tissue culture infectious dose 50% ($TCID_{50}$) of SARS-CoV-2 strain “2019_nCoV Muc-IMB-1” (GISAID ID_EPI_ISL_406862, designation “hCoV-19/Germany/BavPat1/2020”) at 1 mL per nostril, using a vaporization device (Teleflex Medical, Germany). Twenty-four hours after inoculation three contact cattle, that were separated before infection, were re-introduced. Body temperature was monitored daily and nasal, oral and rectal swabs were taken on days −1, 2, 3, 4, 6, 8, 12 and 20, and blood samples on days −1, 6, 12 and 20 after infection. Extensive physical examination was carried out once per day by veterinarians considering parameters as general and feeding behavior, liveliness, body temperature, and posture with a special focus on respiratory disease related signs, such as nasal and ocular discharge, labored breathing, and respiratory sounds. Additionally, for each cattle a daily clinical score has been determined. A value of 0 to 3 points each representing physiologic conditions to severe disease signs was awarded for liveliness, posture, motion and feed intake. Body temperature was also scored: a value of 0 was used for temperatures <39.5°C, 1 for a slightly increased temperature of 39.5° to 40.0°C, 2 for fever between 40.1° and 40.5°C, and a value of 3 for temperatures exceeding 40.5°C. The individual values for each category were summarized, and any animal with a clinical score larger than 3 out of 15 was monitored by a veterinarian at least 3 times a day. Additionally, the cattle were taken care of in the daily morning and evening routine by staff animal caretakers. During the entire study, a clinical score of 0 was calculated for all animals and every day with the exception of animal 771 (scores of 1 two days before infection, 1 one day before infection, and 3 on the day of SARS-CoV-2 inoculation, due to increased body temperature) and the in-contact cattle 774 1 day post co-housing (score of 2 due to an increase in body temperature) (Appendix Figure). Since no other clinical signs were obvious for animal 774, and body temperature dropped below 39.5°C within 8 hours, no further measures were taken. The slight increases in body temperature were not considered to be related to the

SARS-CoV-2 inoculation, as they occurred either before infection or immediately after co-housing.

Sample Processing

Swabs (Medical Wire & Equipment, UK) were immediately resuspended in 1.25 mL serum-free cell culture medium supplemented with penicillin, streptomycin, gentamycin, and amphotericin B. Nucleic acid was extracted from 100µl of swab fluid using the NucleoMag Vet kit (Macherey-Nagel, Germany), and subsequently tested by the real-time RT-PCR "nCoV_IP4" targeting the RNA-dependent RNA polymerase (RdRp) gene (*1*). Positive results were confirmed by a second real-time RT-PCR based on an E gene target (*2*) and a commercial real-time RT-PCR kit (ID GENE SARS-COV-2 DUPLEX, ID.vet, France). Serum samples were tested by indirect immunofluorescence (iIFA) and virus neutralization assays (VNT) against SARS-CoV-2 as described before (*3*), and by an ELISA based on the receptor-binding domain (RBD) of SARS-CoV-2 (K. Wernike, unpub. data, https://www.biorxiv.org/content/10.1101/2020.08.26.266825v1). In addition, the sera were investigated by iIFA using CRFK cells (L0115, collection of cell lines in veterinary medicine (CCLV), Insel Riems) infected with BCoV strain Nebraska as antigen matrix and by VNT against this BCoV strain on MDBK cells (L0261, CCLV).

References

1. World Health Organization. Coronavirus disease (COVID-19) technical guidance: laboratory testing for 2019-nCoV in humans. 2020 [cited 2020 May 16]. https://www.who.int/emergencies/diseases/novel-coronavirus-2019/technical-guidance/laboratory-guidance</eref>

2. Corman VM, Landt O, Kaiser M, Molenkamp R, Meijer A, Chu DKW, et al. Detection of 2019 novel coronavirus (2019-nCoV) by real-time RT-PCR. Euro Surveill. 2020;25:2000045. PubMed https://doi.org/10.2807/1560-7917.ES.2020.25.3.2000045

3. Schlottau K, Rissmann M, Graaf A, Schön J, Sehl J, Wylezich C, et al. SARS-CoV-2 in fruit bats, ferrets, pigs, and chickens: an experimental transmission study. Lancet Microbe. 2020 Jul 7 [Epub ahead of print].

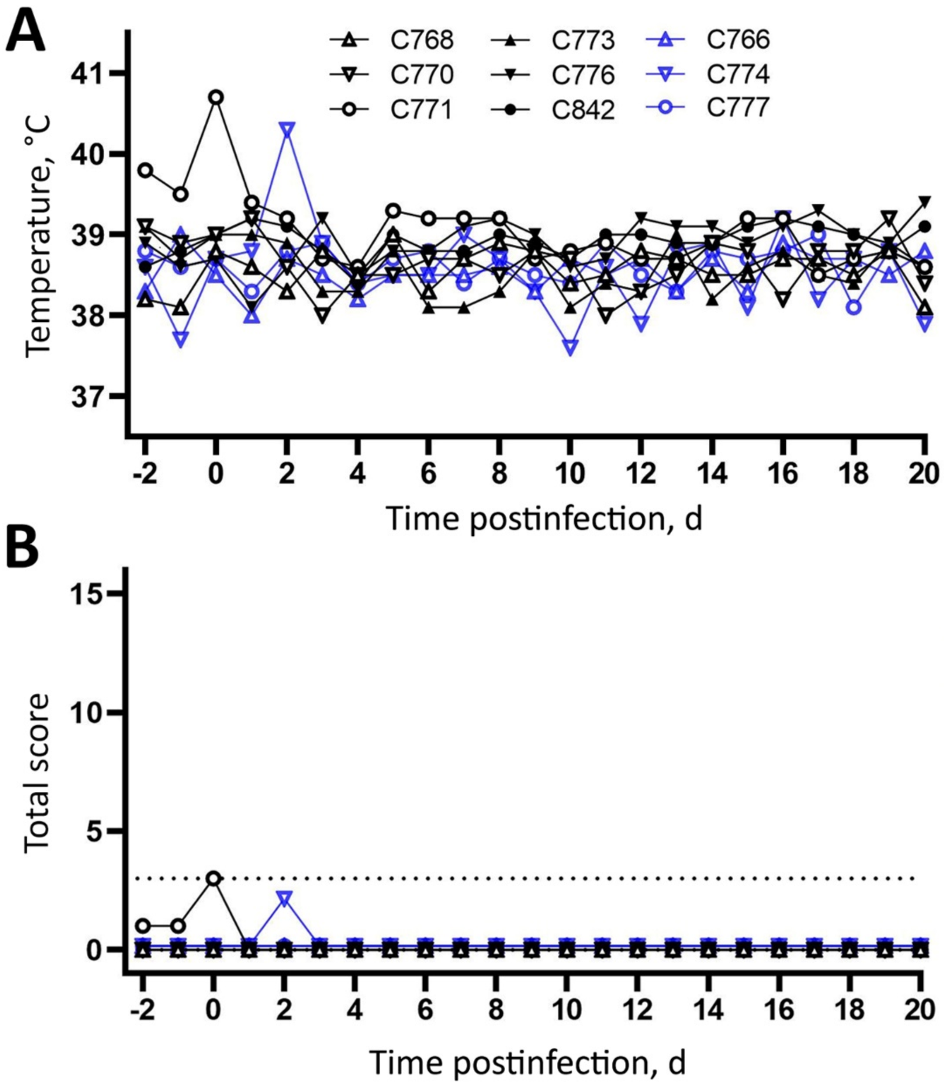

Appendix Figure. Rectal body temperature curves and clinical scores for cattle infected with SARS-CoV-2 and in-contact cattle. Animals directly inoculated are shown in black, while in-contact animals are depicted in blue. A) The daily body temperatures plotted starting 2 days before infection. B) The clinical scores were determined for every animal on each study day and the total score (from 0 to 15) was obtained by summarizing the values awarded for liveliness, posture, motion, feed intake, and body temperature. Any animal with a clinical score >3 (dashed line) was monitored by a veterinarian ≧3 times a day.
